# Cryo-EM demonstrates the in vitro proliferation of an ex vivo amyloid fibril morphology by seeding

**DOI:** 10.1038/s41467-021-27688-5

**Published:** 2022-01-10

**Authors:** Thomas Heerde, Matthies Rennegarbe, Alexander Biedermann, Dilan Savran, Peter B. Pfeiffer, Manuel Hitzenberger, Julian Baur, Ioana Puscalau-Girtu, Martin Zacharias, Nadine Schwierz, Christian Haupt, Matthias Schmidt, Marcus Fändrich

**Affiliations:** 1 Institute of Protein Biochemistry, Ulm University, 89081 Ulm, Germany; 2 Department of Theoretical Biophysics, Max-Planck-Institute of Biophysics, 60438 Frankfurt am Main, Germany; 3 Physics Department T38, Technical University of Munich, 85748 Garching, Germany

**Keywords:** Prions, Protein aggregation, Molecular conformation, Cryoelectron microscopy

## Abstract

Several studies showed that seeding of solutions of monomeric fibril proteins with ex vivo amyloid fibrils accelerated the kinetics of fibril formation in vitro but did not necessarily replicate the seed structure. In this research we use cryo-electron microscopy and other methods to analyze the ability of serum amyloid A (SAA)1.1-derived amyloid fibrils, purified from systemic AA amyloidosis tissue, to seed solutions of recombinant SAA1.1 protein. We show that 98% of the seeded fibrils remodel the full fibril structure of the main ex vivo fibril morphology, which we used for seeding, while they are notably different from unseeded in vitro fibrils. The seeded fibrils show a similar proteinase K resistance as ex vivo fibrils and are substantially more stable to proteolytic digestion than unseeded in vitro fibrils. Our data support the view that the fibril morphology contributes to determining proteolytic stability and that pathogenic amyloid fibrils arise from proteolytic selection.

## Abstract (web summary)

Here, the authors present the cryo-EM structure of in vitro amyloid fibrils from recombinant SAA1.1 protein that were formed by seeding with fibrils purified from systemic AA amyloidosis tissue. This in vitro fibril structure resembles the structure of the ex vivo fibrils but differs from unseeded in vitro fibrils. These findings show that fibril morphologies can be propagated in vitro by seeding.

## Introduction

Amyloid fibrils are fibrillar polypeptide aggregates which occur in a range of diseases that includes several neurodegenerative diseases and different forms of systemic amyloidosis^1,2^. The fibrils can have a length of serval micrometers and consist of cross-β structure and of β-strands that run roughly perpendicular to the fibril main axis^3,4^. Several studies demonstrated that the structure of amyloid fibrils that were extracted from the tissue of patients or animals are structurally different from known in vitro formed fibril structures^5–9^. One example is the fibrils formed from SAA1.1, the fibril precursor protein in systemic AA amyloidosis^7^. Ex vivo amyloid fibrils from SAA1.1 or other proteins differ not only by their structure from in vitro formed fibrils, they are more protease stable^7,8,10^. These observations gave rise to the proteolytic selection hypothesis which assumes that disease-associated amyloid fibrils were selected within the body due to their ability to escape the endogenous proteolysis machinery^7,11^.

The biophysical mechanism of amyloid fibril formation involves a nucleated polymerization mechanism in which the slow initial formation of a fibril nucleus is the rate-limiting step for the fast, subsequent elongation of fibrils^12,13^. Addition of preformed fibrils as seeds to a solution of freshly dissolved fibril precursor protein can overcome the need of de novo nucleation and allows fibril elongation to start immediately^12,13^. The kinetic observation of seeding is commonly thought to involve the replication of the seed structure in the daughter filament, similar to the proliferation mechanism of infectious prions^12,14^. However, this notion was recently challenged by observations that the structure of the daughter fibril is not necessarily the same as the structure of the seed, specifically when using ex vivo fibrils as seeds^8,15,16^.

In the presents study we have analyzed a sample of in vitro seeded amyloid fibrils from recombinant SAA1.1 protein that were formed in the presence of ex vivo AA amyloid fibrils. Systemic AA amyloidosis in mice is one of the best-established cases of a prion-like diseases outside the brain. Since the late 1960ies, evidence has been accumulating that the injection of amyloid-laden tissue extracts into appropriate recipient mice would transfer the disease across animals^17^. In 1982, the year in which Prusiner coined the word ‘prion’^18^, the term ‘amyloid enhancing factor’ (AEF) was published to describe the molecular agent underlying the transmissible activity in systemic AA amyloidosis^19^. In 2002, the Westermark group attributed AEF activity to AA amyloid fibrils and suggested a prion-like mechanism^20^.

**Fig. 1:**
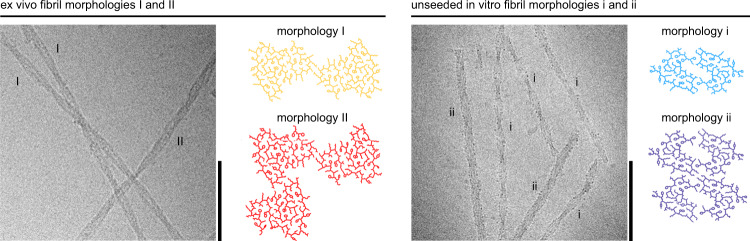
Cryo-EM structures of ex vivo fibrils and of unseeded in vitro fibrils. Cryo-EM images and molecular models of ex vivo fibril morphologies I (orange) and II^7,22^ and of unseeded in vitro fibril morphologies i (light blue) and ii (purple). Scale bar of the cryo-EM images: 100 nm. The micrographs are representative for 1,429 micrographs of the ex vivo and 3,001 of the unseeded in vitro sample. The molecular models show one cross-sectional layer each and are based on the previously deposited PDB files for the ex vivo fibril morphology I (PDB 6DSO), ex vivo fibril morphology II (PDB 6ZCH), unseeded in vitro morphology i (PDB 6ZCF) and unseeded in vitro morphology ii (PDB 6ZCG)^7,22^.

We previously showed that ex vivo amyloid fibrils from AA amyloidotic mice contain two main fibril morphologies^5,21^. Morphology I corresponds to ~95% of these fibrils, while morphology II accounts for most of the remaining fibrils^22^. Structural analysis with cryo-electron microscopy (cryo-EM) revealed that both fibrils contain the same fibril protein fold with ordered conformation at residues Gly1 to Gly69^7^. Morphology I contains two fibril protein stacks (or protofilaments), while morphology II contains an additional third fibril protein stack (or protofilament) (Fig. 1). The fold of ex vivo AA amyloid fibrils differs from the fold of unseeded in vitro fibrils from recombinant SAA1.1 protein (Fig. 1). In vitro fibrils contain at least two fibril morphologies, termed morphologies i and ii, that consist of two or four fibril protein stacks (Fig. 1). The ordered conformation of in vitro fibrils is formed by residues Gly1 to Ala37.

In the present study, we have analyzed as to whether or not these fibril morphologies can be propagated in vitro by seeding recombinant SAA1.1 with ex vivo amyloid fibrils. Our observation that this is indeed the case, at least for morphology I, supports the idea of a proteolytic selection of pathogenic amyloid structures and demonstrates the propagation of proteolytic stability together with a specific fibril morphology.

## Results

### Ex vivo fibrils accelerate the formation of SAA1.1 fibrils in vitro

**Fig. 2:**
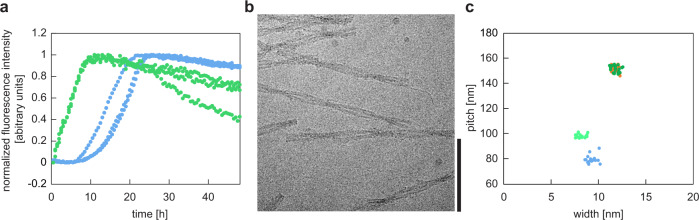
Generation of seeded in vitro fibrils. (**a**) Time-resolved ThT fibrillation kinetics of recombinant SAA1.1 protein without (blue) and with 5% (w/w) ex vivo fibrils as seeds (green). The lag phase of the unseeded reaction is 10.9 ± 1.7 h. (**b**) Cryo-EM image of the seeded in vitro fibrils, showing only fibrils of the major morphology. Scale bar: 100 nm. The micrograph is representative for 1,762 micrographs. (**c**) Cryo-EM based pitch and width measurements with the major (deep green, superimposing with the orange data points) and the minor (light green) fibril morphology of the seeded in vitro fibrils (*n* = 20). Orange: ex vivo fibril morphology I; light blue: unseeded in vitro fibril morphology i. The values of pitch/width are: 151.8 ± 2.5 nm/11.9 ± 0.4 nm (ex vivo I); 151.6 ± 2.5 nm/11.9 ± 0.3 nm (major seeded in vitro); 98.9 ± 1.4 nm/8.2 ± 0.4 nm (minor seeded in vitro); 80.1 ± 3.0 nm/9.3 ± 0.4 nm (unseeded in vitro).

Using the same buffer and solution conditions as in the formation of unseeded in vitro fibrils (fibril formation in a 96-well plate containing 0.2 mg/mL recombinant SAA1.1 in 10 mM Tris buffer, pH 8.5, at 37 °C), we analyzed the effect of 0.01 mg/mL ex vivo fibrils on the kinetics of fibril formation and on the structure of the resulting fibrils. We followed the formation of fibrils in real time based on thioflavin T (ThT) fluorescence (Fig. 2a), which depends on the binding of ThT to amyloid fibrils^23^. These fluorescent properties of ThT are often used to monitor the kinetics of amyloid fibril formation inside a test tube^24^. Without seeds being added, we find that recombinant SAA1.1 protein fibrillates with a well-resolved lag phase of 10.9 ± 1.7 h (Fig. 2a). No lag phase is observed if seeds (ex vivo AA amyloid fibrils) were added at the beginning of the experiment.

**Fig. 3:**
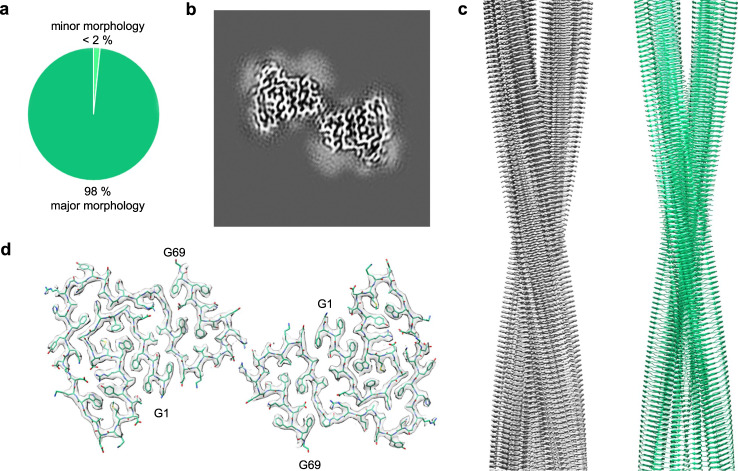
Cryo-EM structure of the major fibril morphology of the seeded in vitro fibrils. (**a**) Relative abundance of the major (deep green) and minor fibril morphology (light green) in the cryo-EM data set (3450 fibrils evaluated). (**b**) 5.2 Å thick slice of the reconstructed density of the major morphology. (**c**) Side view of the reconstructed 3D map (left, grey) of the molecular model (right, light green). (**d**) Cross-sectional view of one molecular layer of the reconstructed density (grey), superimposed with the molecular model (light green).

Based on cryo-EM (Fig. 2b), we observed two fibril morphologies in the sample of seeded fibrils that differed in width and helical pitch (Fig. 2c). The dominating fibril morphology corresponds to 98% of the fibrils in the sample (Fig. 3a) and shows a width of 11.9 ± 0.3 nm and a pitch of 151.6 ± 2.5 nm (Fig. 2c). These structural parameters are highly similar to the ones of ex vivo fibril morphology I (Fig. 2c). The minor morphology corresponds to only 2% of the seeded in vitro fibrils (Fig. 3a) and possesses a width of 8.2 ± 0.4 nm and a helical pitch of 98.9 ± 1.4 nm (Fig. 2c). These parameters do not resemble any of the previously described cryo-EM structures from SAA1.1.

### Cryo-EM demonstrates the proliferation of the seed structure

**Fig. 4:**
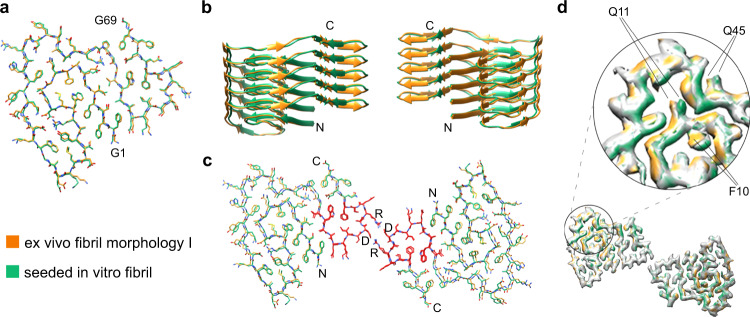
Comparison of the major seeded in vitro fibril and the ex vivo fibril morphology I. **a** Superimposition of the molecular models of the fibril protein in the two fibrils. All atom RMSD: 0.81 Å. **b** Superimposition of ribbon diagrams of five-layer stacks of the two fibrils. **c** Superimposition of one molecular layer of the two fibrils. The all atom RMSD values are 1.09 Å for residues 1–69, 0.46 Å for residues 51–64 (red) and 1.17 Å for residues 1–50 and 65–69. In all three panels: light green, major seeded in vitro fibril; orange, ex vivo fibril. **d** Comparison of the 3D maps of the two fibrils (difference map). The green and orange regions refer to the difference between the two maps.

To clarify its molecular assembly, we obtained the cryo-EM structure of the major seeded in vitro fibril morphology at a resolution of 2.69 Å (Fig. 3b, Supplementary Table 1), based on 0.143 Fourier shell correlation (FSC) criterion (Supplementary Fig. 1a). The resulting molecular model (Fig. 3c, d) possesses a model resolution of 2.6 Å (Supplementary Table 2) and corresponds well to the two-dimensional (2D) classes and power spectra of our cryo-EM images (Supplementary Fig. 1). The fibril contains two protofilaments that are arranged with a pseudo 2_1_ screw symmetry (Fig. 3c). These properties resemble the previously described ex vivo fibril morphology I. The helical parameters (rise 2.4 Å, twist 179.425°, Supplementary Table 1) of the seeded fibril are also very similar to the ex vivo fibril morphology (rise 2.41 Å, twist 179.44°)^22^. The stable structure of the fibril protein extends from Gly1 to Gly69 (Fig. 4a). More C-terminal residues were not seen in the three-dimensional (3D) map, indicating structural disorder.

The fibril protein fold of the seeded in vitro fibril corresponds to the fibril protein fold of ex vivo fibrils. The all atom root mean square deviation (RMSD) is 0.81 Å for the two fibril structures (Fig. 4a). In vitro seeded fibrils and ex vivo fibrils show a similar cross-sectional and z-axial arrangement of the fibril proteins (Fig. 4b, c) with an all atom RMSD of 1.09 Å per molecular layer (Fig. 4c). The greatest similarity occurs at residues 51–64 (RMSD 0.46 Å), while the remaining part of the structures possesses an RMSD of 1.17 Å (Fig. 4c). Therefore, the greatest correspondence occurs at an inner radial position, where the two protofilaments are held together by reciprocal salt bridges between residues D59 and R61 (Fig. 4c). The differences between the two fibril structures are not artefacts of the modeling and observed at the level of the 3D maps as well–specifically at residues Phe10, Gln11 and Gln45 (Fig. 4d).

To test whether the observed variations may be explained by structural fluctuations we performed molecular dynamics (MD) simulations of the two fibril structures. We find that the deviations of the structures before and after simulation for 100 ns (Supplementary Fig. 3a,b) are very similar to the variations between the two cryo-EM structures (Fig. 4c). The two central layers of the simulated fibril segment deviate from the cryo-EM structure by an RMSD value of 3.59 ± 0.05 Å for the ex vivo fibril and of 3.44 ± 0.04 Å for the in vitro seeded fibril before and after simulation. The room mean square fluctuations (RMSF) of the Cα atoms are highest at the terminal ends of the fibril proteins and in a region extending roughly from residue 20 to 40 (Supplementary Fig. 3).

We were not able to obtain a high-resolution reconstruction of the minor seeded in vitro fibril morphology. This fibril is represented in our data set by only 55 images, and the fibril symmetry could not be determined unambiguously. The best reconstruction was obtained by assuming C2 symmetry (Supplementary Fig 5a). The fibril cross-section was very similar to morphology i of the unseeded in vitro fibrils (Supplementary Fig. 2), which possesses a pseudo-2_1_ screw symmetry^7^. This fibril differs from the presently studied one by showing a different overall topology in the original cryo-EM images and differences in the fibril pitch (Fig. 2c). We conclude that the fibril protein fold of the minor seeded in vitro fibril is very similar, if not identical, to the protein fold of the unseeded in vitro fibrils. However, the two fibril differs in topological properties, possibly including the fibril symmetry.

### Seeding confers proteolytic stability to the daughter fibrils

**Fig. 5:**
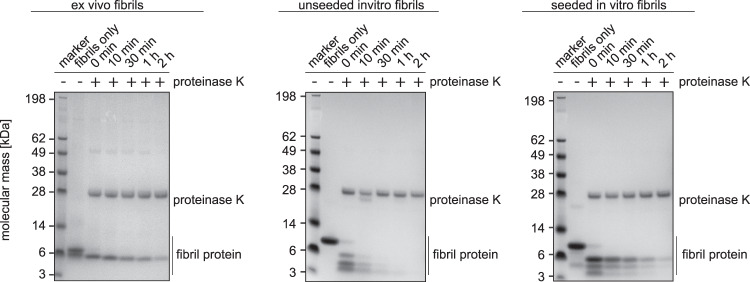
Seeding propagates the high proteolytic resistance of ex vivo fibrils. Coomassie stained denaturing protein electrophoresis gels with seeded and unseeded in vitro fibrils, as well as with ex vivo fibrils, which were incubated with proteinase K for different periods of time as indicated in the figure. The lane next to the marker shows fibrils before proteinase K addition. All experiments were performed in triplicates (*n* = 3).

Based on that proteolytic resistance constitutes a key difference between ex vivo amyloid fibrils and in vitro formed amyloid fibrils, including the in vitro and ex vivo fibrils from SAA1.1 protein^7,8,10,11^, we tested the susceptibility of the different fibril samples to proteolytic digestion. Subjecting samples of the seeded and unseeded in vitro fibrils, as well as of ex vivo AA amyloid fibrils to protease K digestion (Fig. 5) we find ex vivo fibrils to be substantially more protease resistant than the unseeded in vitro fibrils (Fig. 5), fully reproducing our previous observations^7^. Seeded in vitro fibrils were also stable to proteolytic digestion and persisted for more than 2 h under the conditions of our experiment, similar to the ex vivo fibrils (Fig. 5). Hence, proteolytic resistance arises, at least partly, from the fold of the fibril protein.

Support for this view comes from a second set of experiment in which we subjected in vitro seeded and unseeded fibrils to a digestion with pronase E (Supplementary Fig. 6). Similar to the use of proteinase K we find that the seeded fibrils are more proteolytically stable than unseeded fibrils. While unseeded fibrils are completely digested, a pronase E stable fragment persists in the seeded in vitro fibrils until the end of the experiment (120 min). This fragment migrates immediately below the 6 kDa marker band, similar to the pronase E stable fragment of ex vivo fibrils^7^ and the proteinase K stable fragment of ex vivo fibrils and seeded in vitro fibrils (Fig. 5). However, it is possible that seeded in vitro fibrils are slightly less stable than ex vivo fibrils as there is a transient formation of low molecular weight fragments at 3-6 kDa that resemble the transient proteolytic fragments obtained with unseeded in vitro fibrils (Fig. 5). Upon pronase E treatment, however, such fragments are not very abundant on the gel (Supplementary Fig. 6).

## Discussion

In this research we show that the addition of ex vivo AA amyloid fibrils to a sample of freshly dissolved recombinant SAA1.1 protein induces the formation of an amyloid fibril structure that corresponds to the main morphology of the ex vivo fibrils (Fig. 4). The structural correspondence extends from the protein fold (Figs. 3a,  4a), over the arrangement of the fibril proteins into two protofilaments (Fig. 4b, c) to the helical symmetry and the resistance of the fibrils to proteinase K digestion (Fig. 5). While we noted small structural differences between the two fibril structures, they probably arise from structural fluctuations, resembling the dynamic structural fluctuations of microtubules^25,26^. Seeding proliferated only the structure of the major but not of the minor ex vivo fibril to the daughter fibrils. The loss of the minor morphology, which contains three protofilaments (Fig. 1), and the increase of the main morphology from 95% in the ex vivo fibrils^22^ to 98% in the in vitro seeded fibrils (Fig. 3a) implies that the main morphology, which contains two protofilaments, is kinetically favored.

Our observations contrast to several recent studies that indicated that the ex vivo fibril structure is not necessarily replicated by seeding in vitro^15,27,28^ To analyze the possible basis of these different findings we analyzed a number of factors that define the conditions of fibril formation, that are generally known to determine the fibril morphology^29^. We compared the concentration of the monomeric fibril protein, the concentration of the ex vivo fibrils relative to the monomeric protein, and the purity of the ex vivo fibrils in the different studies that likely affect the competition between (unwanted) de novo nucleation and (wanted) seed extension reactions. That is, seed extension is favored by high relative seed concentrations, while unwanted de novo nucleation reactions are unfavorable at low monomeric protein concentrations and when using pure fibril seeds that do not nucleate alternative assembly pathways. Consistent with this view, the molar concentration of recombinant SAA1.1 protein used in our study (~17 µM) is lower than the molar concentrations used in the previous studies (50–100 µM)^15,27,28^, and the relative seed concentration is 5% (w/w) in our study and 2.5 to 5% in the previous studies, although the relative seed concentration was not always reported^15,27^. Our seeds lacked major protein contaminants (Fig. 5), while previous studies did not report the seed purity^15,27,28^.

These considerations might indicate that our experimental conditions were more favorable to seed extension than in the other studies, but the three aforementioned factors depend on protein-specific features and are thus difficult to compare. Moreover, the chemical composition of the fibrillation reaction (buffer molecule, ions etc.) may also affect the fibril morphology and in the case of ex vivo α-synuclein fibrils there is evidence that a molecular cofactor is an integral part of the fibril structure, suggesting that the in vitro seeded fibrils did not fully replicate the ex vivo fibril structure if this molecular component was missing from the fibrillation reaction^15^. Nevertheless, there is evidence that a small fraction of the SAA1.1 protein in our samples was not recruited into morphology I and instead adopted a fibril protein conformation that strongly resembled the one in unseeded in vitro fibrils (Supplementary Fig. 5c). This fraction of the fibril protein must have undergone a de novo fibril nucleation reaction to generate a fibril structure that is different from the added seeds.

Our data help to improve our understanding of the mechanism of amyloid fibril formation from SAA1.1 inside the body and provide new evidence on the long-standing debate as to what comes first: fibril formation or the proteolytic truncation of the fibril protein. SAA1.1 is C-terminally truncated in the ex vivo fibrils, mainly at residues 75–83^20,22^. As the N-terminus of SAA1.1 is the most aggregation prone segment of the protein^30,31^, it seems possible that C-terminal truncations could generate a highly amyloidogenic fragment that constitutes the direct precursor of the fibrils, similar to the generation of Aβ peptide in Alzheimer’s disease^1^. However, the high exposure of the fibril protein C-terminus in the structure of the ex vivo AA amyloid fibrils and its conformational disorder^7,22^ indicated that proteolysis may have occurred after the formation of fibrils, and to type A transthyretin fibrils in systemic ATTR amyloidosis^32^. Indeed, our observations that full-length SAA1.1 can be sequestered into morphology I supports this view and demonstrates that SAA1.1 does not need to be truncated in order to assemble into this fibril state.

A second ramification for fibril formation in vivo arises from the high proteolytic stability of the in vitro seeded fibrils. Proteolytic stability was previously found to characterize ex vivo amyloid fibrils^8,10^ and it was suggested to be key for the formation of pathogenically relevant^7,11^. Our observations support this concept by showing that proteolytic stability can be proliferated upon seeding. These data imply that proteolytic stability depends, at least partly, on the fibril morphology. Interestingly, SAA2.2 protein, which prevents the development of amyloidosis in CE/J mice^33^, was shown to form fibrils in vitro that were unstable in urea and denatured upon raising the temperature^34^. Hence, SAA2.2 might be non-pathogenic because the instability of its fibrils prevents them from contributing to disease.

The present work and several previous studies demonstrate that systemic AA amyloidosis is an excellent model system for studying phenomena that are relevant for a broad range of protein misfolding diseases. Prion-like features are observed in cell or animal models of many protein misfolding diseases and may underlie the staging of these diseases and the spreading of the amyloid deposits throughout an organ or throughout the body^14^. Our present observations open the door to a broad range of possible follow up studies to investigate the kinetics and the mechanism of ex vivo-like fibril proliferation with detailed biophysical studies. They are further relevant for attempts to obtain fibrils with an ex vivo-like structure inside the test tube, for example, as a basis of structural studies with nuclear magnetic resonance spectroscopy^27,28^.

## Methods

### Recombinant protein expression and purification

Murine full-length SAA1.1 protein was recombinantly expressed in the *Escherichia coli* RV308 cells as described previously^35^. In brief, the SAA1.1 coding region was cloned in the pMAL-c2X vector (New England Biolabs) at the C-terminus of a His-tagged maltose-binding protein, which carried a cleavage site for tobacco etch virus protease. Protein purification was carried out by the following five steps: (i) chromatography via amylose resin high flow (New England Biolabs), applying a linear gradient of 0 to 10 mM maltose in tris(hydroxymethyl)aminomethane (Tris) buffer A [20 mM Tris/HCl, pH 7.5, 200 ml NaCl], (ii) chromatography via nickel-sepharose fast flow resin (Cytiva), applying a linear gradient of 0 to 250 mM imidazol in Tris buffer B [20 mM Tris/HCl, pH 8.0, 150 mM NaCl], (iii) overnight fusion protein cleavage using tobacco etch virus protease at 34 °C, (iv) chromatography via nickel-sepharose fast flow resin using the same conditions as in step (ii) to separate SAA1.1 from the His-tagged maltose-binding protein, (v) chromatography via Source 15 RPC reversed-phase medium (GE Healthcare), applying a linear gradient from 0 to 86% (v/v) acetonitrile in 0.1% (v/v) trifluroacetate. The purified protein was lyophilized with an alpha 2-4 LD plus freeze dryer (Christ).

### Fibril extraction from tissue

AA amyloidotic NMRI mice (Charles River Laboratories) were obtained as described previously^22^. The animals were generated based on an animal experiment permission (no. 1165) from the Regierungspräsidium Tübingen. The mice used in the study had the following housing conditions: temperature: 22.4 °C, humidity: 57%, dark/light cycle: 12 h dark/12 h light. AA amyloid fibrils were extracted from an amyloid-laden mouse liver based on a previously described extraction protocol^5^. In brief, 100 mg of tissue material were washed five times with 1 mL Tris Calcium Buffer [20 mM Tris, 138 mM NaCl, 2 mM CaCl_2_, 0.1% (w/v) NaN_3_, pH 8.0]. Samples were centrifuged at 3100 × g for 1 min at 4 °C. The pellet was resuspended in 1 mL freshly prepared collagenase/protease inhibitor solution [one protease inhibitor ethylenediamine-tetraacetic acid (EDTA)-free tablet (cOmplete^TM^, Roche) in 7 mL Tris Calcium Buffer, 5 mg/mL crude collagenase from *Clostridium hitolyticum* (Sigma)] and incubated overnight at 37 °C at 250 rpm in an IKA MTS 2/4 digital table shaker. Afterwards, the tissue material was centrifuged at 3100 × g for 30 min at 4 °C. The pellet was resuspended in 1 mL Tris EDTA Buffer [20 mM Tris, 140 mM NaCl, 10 mM EDTA, 0.1% (w/v) NaN_3_, pH 8.0] and homogenized. The homogenate was centrifuged for 5 min at 3100 × g at 4 °C. This step was repeated two times. Afterwards, the tissue pellet was homogenized in 200 μL ice cold water. The homogenate was centrifuged for 5 min at 3100 × g at 4 °C and the fibril containing supernatant was stored. This step was repeated four times.

### Formation of seeded in vitro fibrils

Recombinant murine SAA1.1 was incubated at 0.2 mg/mL in 10 mM Tris buffer, pH 8.5, for 48 h at 37 °C. Seeded samples additionally contained 0.01 mg/mL ex vivo fibrils extracted from murine tissue which corresponds to 5% (w/w) relative to the recombinant SAA1.1. Incubation was carried out in a black 96-well plate (Greiner Bio-One) at 37 °C in a FLUOstar OMEGA plate reader (BMG Labtech). The sample volume in each well was 100 µL and the plate was agitated every 30 min by double orbital shaking for 10 s at 100 rpm.

### Fibrillation kinetics measurements

Time-resolved ThT fluorescence measurements were carried out in a 96-well plate (Greiner Bio-One, 96 F-Bottom) at 37 °C using a FLUOstar Omega plate reader (BMG Labtech) at an excitation wavelength of 450 nm, an emission wavelength of 490 nm, 20 flashes/well, every 30 min over a period of 48 h. Before each measurement the sample was agitated by orbital shaking for 10 s at 100 rpm. All samples had a volume of 100 µL and contained 0.2 mg/mL freshly dissolved SAA1.1 protein, 10 mM Tris buffer (pH 8.5) and 20 µM ThT. The seeded samples additionally contained 0.01 mg/mL ex vivo AA amyloid fibrils extracted from murine tissue.

### Cryo-EM

A 3.5 µL aliquot of the seeded in vitro fibrils was applied to glow-discharged holey carbon coated grids (400 mesh C-flat 1.2/1.3), blotted with filter paper and plunge-frozen in liquid ethane using a Vitrobot Mark 3 (Thermo Fisher Scientific). Grids were screened using a JEM-2100 transmission electron microscope (Jeol) at 200 kV. Images were acquired using a K2-Summit detector (Gatan) in counting mode on a Titan Krios transmission electron microscope (Thermo Fisher Scientific) at 300 kV. Data acquisition parameters are listed in Supplementary Table 1.

### Helical reconstruction

Movie frames were corrected for gain reference using IMOD^36^. Motion correction and dose-weighting was done using MOTIONCOR 2.1^37^. The contrast transfer function was estimated from the motion-corrected images using Gctf^38^. Helical reconstruction was performed using RELION 3.0.4^39^. Fibrils of each morphology were picked manually. Segments were extracted according to Supplementary Table 1. Reference-free 2D classification with a regularization value of T = 2 was used to select class averages showing the helical repeat along the fibril axis. As initial 3D model a featureless cylinder was used that was created by using relion_helix_toolbox which is implemented in RELION. The resulting reconstructions showed clearly separated β-sheets (*x*–*y* plane) and partially resolved β-strands along the fibril axis. The generated primary model indicated the presents of two identical protein stacks, related by a pseudo-2_1_ screw symmetry. Imposing these symmetries during reconstruction yielded clearly separated β-strands and side-chain densities. 3D classification with local optimization of helical twist and rise was used to further select particles in the in vitro fibril data set for a final high-resolution auto-refinement. The best 3D classes were selected manually and reconstructed with local optimization of helical parameters using 3D auto-refinement. All 3D classification and auto-refine processes were carried out using a central part of 10% of the intermediate asymmetrical reconstruction. The final reconstructions were post-processed with a soft-edge mask and B-factor sharpened. The resolutions of the individual reconstructions were estimated from the FSC at 0.143 between two independently refined half-maps.

### Model building

The model was built by using the previously described ex vivo fibril morphology I^22^ as a starting model. The structural refinement was done with Coot^40^ as well as phenix.real_space_refine^41^ with non-crystallographic symmetry constraints, Ramachandran, atomic displacement parameter and rotamer restraints. Manually defined beta sheets were used as secondary structure restraints. The atomic clashes, rotamer and Ramachandran outliers and model geometry were analyzed by the validation output generated using MolProbity^42^ and the comprehensive validation tool in Phenix^41^. Once a satisfactory main and side-chain density fit was achieved for one polypeptide chain, a fibril stack comprising twelve poly-peptide chains was assembled using the pdbsymm tool implemented in Situs^43^. The described process of iterative refinement and modeling was repeated for the fibril stack over and over again, until the refinement converged to produce reasonable density to model fit. The structural statistics for refinement and model building are listed in Supplementary Table 2.

### Proteolytic digest of fibrils

Proteinase K: a 180 µl aliquot from a stock solution of seeded or unseeded in vitro fibrils or of ex vivo fibrils (0.2 mg/ml each) was mixed with 20 µl 200 mM Tris buffer, pH 8.0, containing 1.4 M NaCl, 20 mM CaCl_2_ and 0.4 µl proteinase K (20 mg/ml, Fermentas). The mixture was incubated at 37 °C and 20 µl aliquots were taken after 0, 10, 30, 60 and 120 min. Proteinase K activity was stopped by adding 0.5 µL of 200 mM PMSF solution that was dissolved in methanol. After 10 min of incubation the samples was frozen in liquid nitrogen until they were analyzed. The proteolytic digestion products were analyzed using denaturing protein gel electrophoresis.

Pronase E: a 90 µl aliquot from a stock solution of seeded and unseeded in vitro fibrils (0.2 mg/ml each) was mixed with 5 µl 2 M Tris buffer, pH 7.5 and 5 µl pronase E from *Streptomyces griseus* (0.8 mg/ml, Sigma-Aldrich). The samples were incubated at 37 °C and 15 µl aliquots were taken after 0, 10, 30, 60 and 120 min. The pronase E activity was stopped by adding 2.25 µl cOmplete EDTA-free protease inhibitor cocktail (Roche) that was prepared by dissolving 1 tablet in 7 ml Millipore water. After 10 min of incubation at room temperature, the samples were plunge frozen in liquid nitrogen until they were analyzed using denaturing protein gel electrophoresis.

### Denaturing gel electrophoresis

A 4–12% NuPAGE Bis-Tris gel (Thermo Fischer Scientific) was loaded with fibril samples to be analyzed that had been mixed with 4× NuPAGE LDS Sample Buffer (Thermo Fischer Scientific) at 3:1 ratio and incubated at 95 °C for 10 min in a heating block. SeeBlue Plus2 prestained protein marker (Thermo Fischer Scientific) was loaded into the gel. After electrophoresis the gels were stained for 1 h at room temperature with Coomassie staining solution comprising 2.5% (w/v) Coomassie Brilliant Blue R250, 30% (w/v) ethanol and 10% (v/v) acetic acid and destained using 20% (v/v) ethanol, 10% (v/v) acetic acid solution (source data are provided as Source Data file).

### MD simulation

We used all-atom MD simulations to characterize the conformational dynamics of the seeded in vitro fibril (PDB 7OVT) and the ex vivo fibril morphology I (PDB 6DSO). Each fibril was simulated as a six-layered stack, consisting of twelve 69-residue fibril proteins. The simulation box had a size of 156.89 Å in all directions and was filled with 123,415 water molecules. The system was neutralized with 0.15 M NaCl, leading to a system size of 506,810 atoms. The force-field parameters for the peptides were taken from Amber99sb-star-ildn^44^. TIP4P-Ew^45^ was used for the water molecules. For NaCl, we used the Mamatkulov-Schwierz force field parameters^46^. The MD simulations were performed at fixed particle number, pressure and temperature using the Gromacs simulation package, version 2018^47,48^. Periodic boundary conditions were applied, and the particle-mesh Ewald method was used for the periodic treatment of Coulombic interactions. Bonds to hydrogen atoms were constrained using a linear constraint solver, and a 2 fs time step was used. To equilibrate the system, we first performed an energy minimization with the steepest descent algorithm. Both systems were equilibrated for 1 ns, first in the canonical ensemble using a constant amount of substance, volume and temperature and then in the isothermal-isobaric ensemble in which the amount of substance, pressure and temperature are conserved. Finally, a 100 ns MD simulation was carried out each, employing the velocity rescaling thermostat with stochastic term, with a time constant of 0.1 s^−1^, and isotropic Parrinello−Rahman pressure coupling, with a time constant of 5 s^−1^. The RMSF was calculated from the production run discarding the first 10 ns for equilibration.

### RMSD value calculation

All-atom RMSDs of single layers have been calculated with VMD 1.9.3^49^ following structural alignment of all atoms in the layer. Values for MD simulations are given as averages of RMSDs that have been obtained for the two central layers in the computer model.

### Morpological analysis and image representation

Morphological anaylsis were obtained by visual inspection of cryo-EM images. Measurements of fibril width and pitch were carried out by using Fiji^50^. Image representations of reconstructed densities and refined models were created by using UCSF Chimera^51^.

### Reporting summary

Further information on research design is available in the Nature Research Reporting Summary linked to this article.

## Supplementary information

Supplementary Information

Reporting Summary

## Data Availability

The reconstructed cryo-EM map was deposited in the Electron Microscopy Data Bank (EMDB) with the accession codes EMD-13089. The coordinates of the fitted atomic model were deposited in the Protein Data Bank (PDB) under the accession code 7OVT. The following previously published coordinates were used in Fig. 1: PDB 6DSO^22^, 6ZCH, 6ZCF, 6ZCG^7^; Fig. 4: PDB 6DSO^22^; Supplementary Fig. 4: PDB 6ZCF^7^. The source data associated with following figures has been provided with this paper: Figs. 2, 3, 5, Supplementary Fig. 1. The data that support the findings of this study are available from the corresponding author upon reasonable request. Source data are provided with this paper.

## Source data

Source Data

## References

[1] Benson MD (2020). Amyloid nomenclature 2020: update and recommendations by the International Society of Amyloidosis (ISA) nomenclature committee. Amyloid.

[2] Chiti F, Dobson CM (2017). Protein misfolding, amyloid formation, and human disease: a summary of progress over the last decade. Annu. Rev. Biochem..

[3] Riek R, Eisenberg DS (2016). The activities of amyloids from a structural perspective. Nature.

[4] Fändrich M (2007). On the structural definition of amyloid fibrils and other polypeptide aggregates. Cell. Mol. Life Sci..

[5] Annamalai K (2017). Common fibril structures imply systemically conserved protein misfolding pathways in vivo. Angew. Chem. - Int. Ed..

[6] Zhang W (2019). Heparin-induced tau filaments are polymorphic and differ from those in alzheimer’s and pick’s diseases. Elife.

[7] Bansal A (2021). AA amyloid fibrils from diseased tissue are structurally different from in vitro formed SAA fibrils. Nat. Commun..

[8] Kollmer M (2019). Cryo-EM structure and polymorphism of Aβ amyloid fibrils purified from Alzheimer’s brain tissue. Nat. Commun..

[9] Schweighauser M (2020). Structures of α-synuclein filaments from multiple system atrophy. Nature.

[10] Schönfelder, J. et al. Protease resistance of ex vivo amyloid fibrils implies the proteolytic selection of disease-associated fibril morphologies. *bioRxiv* 2021.07.05.451219 (2021) 10.1101/2021.07.05.451219. 10.1080/13506129.2021.1960501 34338090

[11] Fändrich M, Schmidt M (2021). Methods to study the structure of misfolded protein states in systemic amyloidosis. Biochem. Soc. Trans..

[12] Jarrett JT, Lansbury PT (1993). Seeding ‘one-dimensional crystallization’ of amyloid: a pathogenic mechanism in Alzheimer’s disease and scrapie?. Cell.

[13] Knowles TPJ, Vendruscolo M, Dobson CM (2014). The amyloid state and its association with protein misfolding diseases. Nat. Rev. Mol. Cell Biol..

[14] Jucker M, Walker LC (2013). Self-propagation of pathogenic protein aggregates in neurodegenerative diseases. Nature.

[15] Lövestam S (2021). Seeded assembly in vitro does not replicate the structures of α-synuclein filaments from multiple system atrophy. FEBS Open Bio.

[16] Radamaker L (2021). Cryo-EM reveals structural breaks in a patient-derived amyloid fibril from systemic AL amyloidosis. Nat. Commun..

[17] Kisilevsky R (1995). Arresting amyloidosis in vivo using small-molecule anionic sulphonates or sulphates: implications for Alzheimer’s disease. Nat. Med..

[18] Prusiner SB (1982). Novel proteinaceous infectious particles cause scrapie. Science.

[19] Axelrad MA, Kisilevsky R, Willmer J, Chen SJ, Skinner M (1982). Further characterization of amyloid-enhancing factor. Lab. Investig..

[20] Lundmark K (2002). Transmissibility of systemic amyloidosis by a prion-like mechanism. Proc. Natl. Acad. Sci. U. S. A..

[21] Annamalai K (2016). Polymorphism of amyloid fibrils in vivo. Angew. Chem. - Int. Ed..

[22] Liberta F (2019). Cryo-EM fibril structures from systemic AA amyloidosis reveal the species complementarity of pathological amyloids. Nat. Commun..

[23] Levine H (1995). Thioflavine t interaction with amyloid βsheet structures. Amyloid.

[24] Buell AK, Dobson CM, Knowles TPJ, Welland ME (2010). Interactions between Amyloidophilic dyes and their relevance to studies of amyloid inhibitors. Biophys. J..

[25] Manka SW, Moores CA (2018). Microtubule structure by cryo-EM: snapshots of dynamic instability. Essays Biochem..

[26] Debs GE, Cha M, Liu X, Huehn AR, Sindelar CV (2020). Dynamic and asymmetric fluctuations in the microtubule wall captured by high-resolution cryoelectron microscopy. Proc. Natl Acad. Sci. U. S. A.

[27] Pradhan T (2020). Seeded fibrils of the germline variant of human l-III immunoglobulin light chain FOR005 have a similar core as patient fibrils with reduced stability. J. Biol. Chem..

[28] Lu JX (2013). Molecular structure of β-amyloid fibrils in alzheimer’s disease brain tissue. Cell.

[29] Fändrich M (2018). Amyloid fibril polymorphism: a challenge for molecular imaging and therapy. J. Intern. Med..

[30] Westermark GT, Engström U, Westermark P (1992). The N-terminal segment of protein AA determines its fibrillogenic property. Biochem. Biophys. Res. Commun..

[31] Rennegarbe M, Lenter I, Schierhorn A, Sawilla R, Haupt C (2017). Influence of C-terminal truncation of murine Serum amyloid A on fibril structure. Sci. Rep.

[32] Schmidt M (2019). Cryo-EM structure of a transthyretin-derived amyloid fibril from a patient with hereditary ATTR amyloidosis. Nat. Commun..

[33] Sipe JD (1993). Characterization of the inbred CE/J mouse strain as amyloid resistant. Am. J. Pathol..

[34] Ye Z (2011). Inflammation protein SAA2.2 spontaneously forms marginally stable amyloid fibrils at physiological temperature. Biochemistry.

[35] Claus S (2017). Cellular mechanism of fibril formation from serum amyloid A1 protein. EMBO Rep..

[36] Kremer JR, Mastronarde DN, McIntosh JR (1996). Computer visualization of three-dimensional image data using IMOD. J. Struct. Biol..

[37] Zheng SQ (2017). MotionCor2: anisotropic correction of beam-induced motion for improved cryo-electron microscopy. Nat. Methods.

[38] Zhang K (2016). Gctf: real-time CTF determination and correction. J. Struct. Biol..

[39] Zivanov J (2018). New tools for automated high-resolution cryo-EM structure determination in RELION-3. Elife.

[40] Emsley P, Lohkamp B, Scott WG, Cowtan K (2010). Features and development of Coot. Acta Crystallogr. Sect. D. Biol. Crystallogr..

[41] Liebschner D (2019). Macromolecular structure determination using X-rays, neutrons and electrons: recent developments in Phenix. Acta Crystallogr. Sect. D. Struct. Biol..

[42] Williams CJ (2018). MolProbity: more and better reference data for improved all-atom structure validation. Protein Sci..

[43] Wriggers W (2012). Conventions and workflows for using Situs. Acta Crystallogr. Sect. D. Biol. Crystallogr..

[44] Lindorff-Larsen K (2010). Improved side-chain torsion potentials for the Amber ff99SB protein force field. Proteins Struct. Funct. Bioinforma..

[45] Horn HW (2004). Development of an improved four-site water model for biomolecular simulations: TIP4P-Ew. J. Chem. Phys..

[46] Mamatkulov S, Schwierz N (2018). Force fields for monovalent and divalent metal cations in TIP3P water based on thermodynamic and kinetic properties. J. Chem. Phys..

[47] Van Der Spoel D (2005). GROMACS: fast, flexible, and free. J. Comput. Chem..

[48] Hess B, Kutzner C, Van Der Spoel D, Lindahl E (2008). GRGMACS 4: algorithms for highly efficient, load-balanced, and scalable molecular simulation. J. Chem. Theory Comput..

[49] Humphrey W, Dalke A, Schulten K (1996). VMD: vsual molecular dynamics. J. Mol. Graph..

[50] Schindelin J (2012). Fiji: An open-source platform for biological-image analysis. Nat. Methods.

[51] Pettersen EF (2004). UCSF Chimera–A visualization system for exploratory research and analysis. J. Comput. Chem..

