# Supplementary material for: Cryo-EM demonstrates the in vitro proliferation of an ex vivo amyloid fibril morphology by seeding
Source: Nat Commun. 2022 Jan 10;13:85. doi: 10.1038/s41467-021-27688-5 (PMC8748726; doi:10.1038/s41467-021-27688-5)
Supplement: Supplementary file 1 — Supplementary Information [file 41467_2021_27688_MOESM1_ESM.pdf]

Supplementary Information

Cryo-EM demonstrates the in vitro proliferation of an ex vivo amyloid fibril morphology by seeding

Thomas Heerde, Matthies Rennegarbe, Alexander Biedermann, Dilan Savran, Peter B. Pfeiffer, Manuel Hitzenberger, Julian Baur, Ioana Puscalau-Girtu, Martin Zacharias, Nadine Schwierz, Christian Haupt, Matthias Schmidt, Marcus Fändrich

Supplementary Figures

Supplementary Figure 1

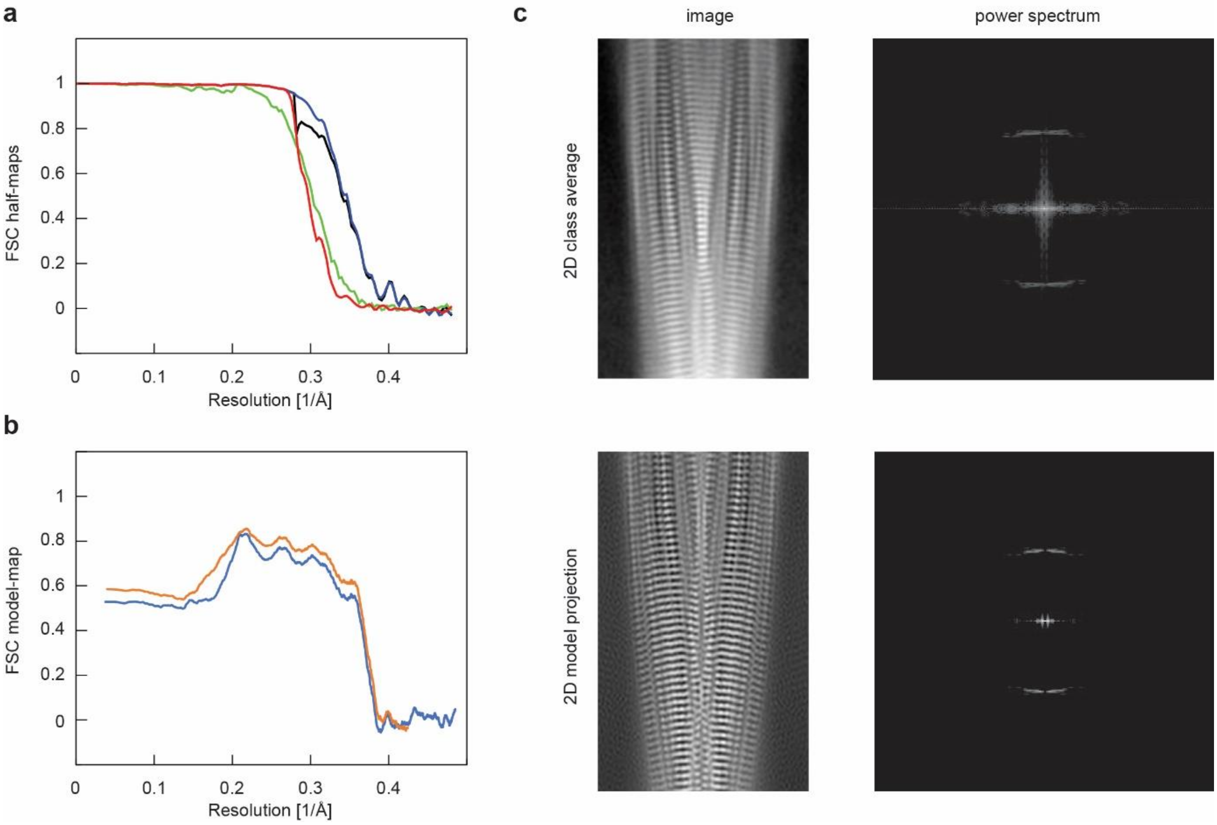

Supplementary Figure 1.

Consistency of the molecular model with the reconstructed 3D map.

(a) FSC of the two half maps. Black: FSC corrected; green: FSC unmasked maps; blue: FSC masked maps; red: corrected FSC phase randomized masked maps. (b) Model-map FSC. Blue: FSC unmasked maps; orange: FSC masked maps. (c) Power spectrum and 2D class average of a consistent part of the fibril (top) and model density projection and power spectrum hereof from of the same fibril region.

Supplementary Figure 2

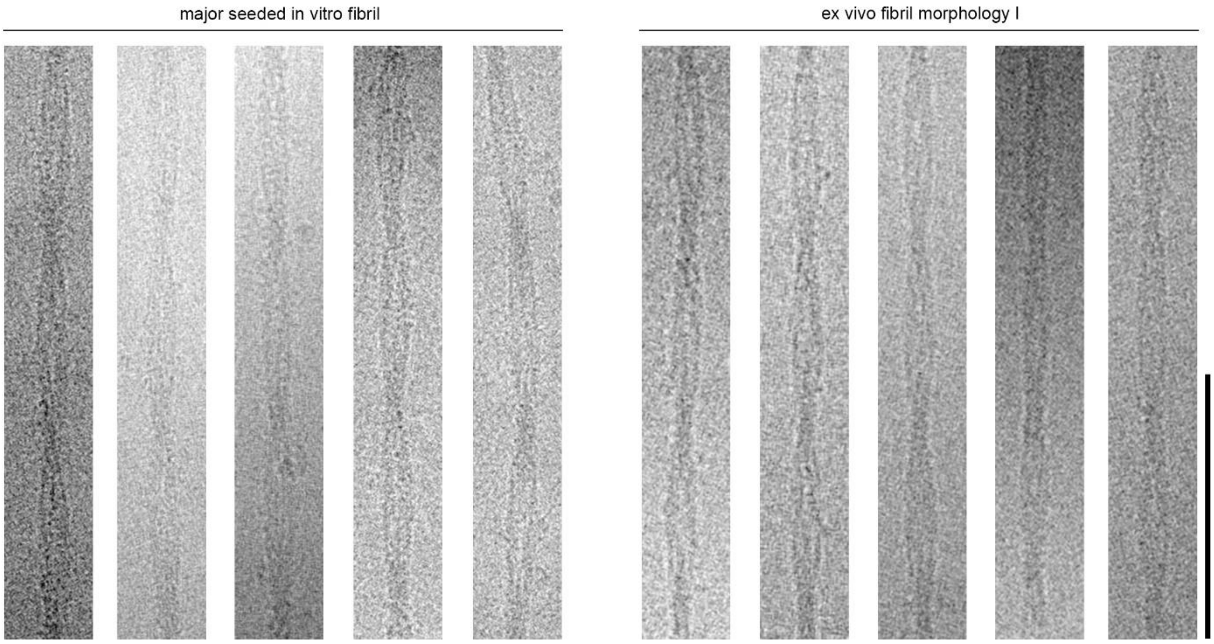

Supplementary Figure 2.

Cryo-EM images of the major morphology of the seeded in vitro fibrils and of ex vivo fibril morphology I.

The cryo-EM images are very similar for the two fibrils. Scalebar: 100 nm. The micrographs are representative for 1,429 micrographs of the ex vivo and 3,001 of the in vitro sample.

Supplementary Figure 3

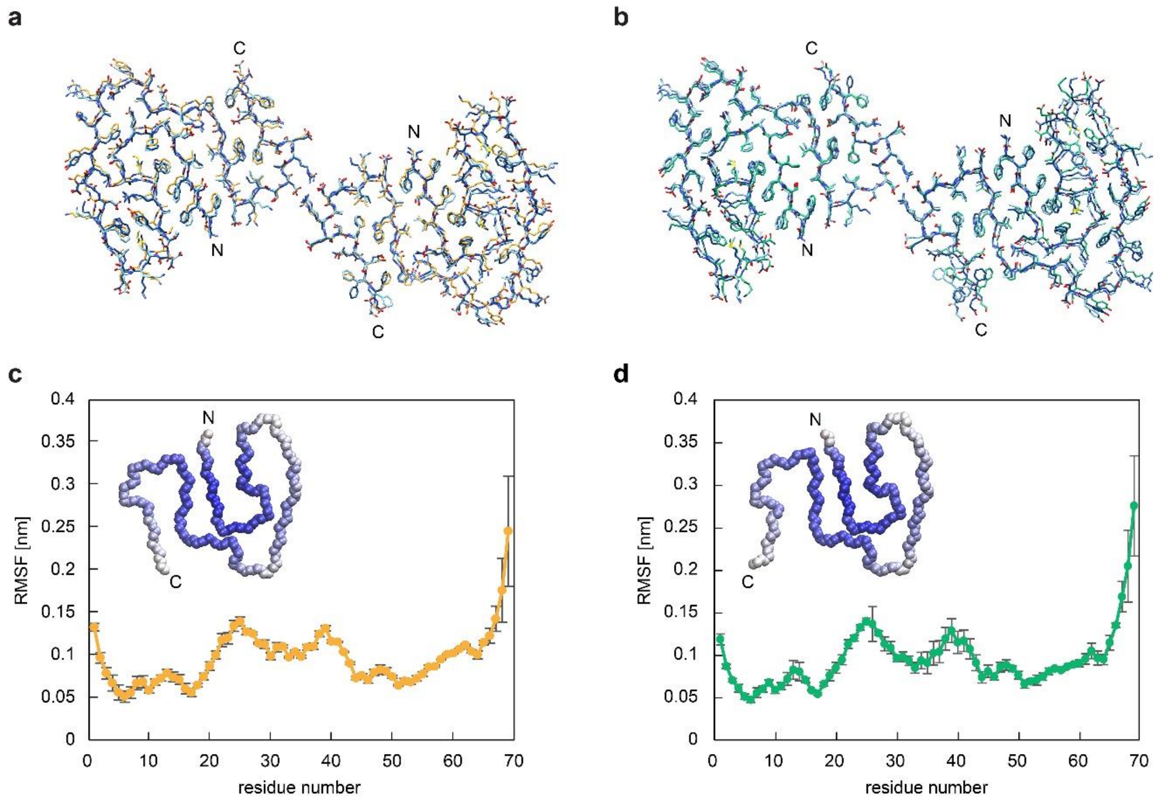

Supplementary Figure 3

MD simulations of the two fibril structures.

(a, b) Superimposition of one molecular layer of ex vivo fibril morphology I (a, 6DSO, orange) and of the major seeded in vitro fibril morphology (b, 7OVT, green) with the two central layers of the respective MD simulations after 100 ns (different shades of blue). (c, d) Mean RMSF values of the Cα atoms of the central two layers of MD simulations of ex vivo fibril morphology I (c) and of the major seeded in vitro fibril (d). The plotted RMSF values are averages over all four fibril protein molecules (n=4) within the two central layers, while error bars refer to the standard deviation. The structures shown represent one polypeptide chain of the respective simulated stack where a deeper shade of blue represents a lower RMSF.

Supplementary Figure 4

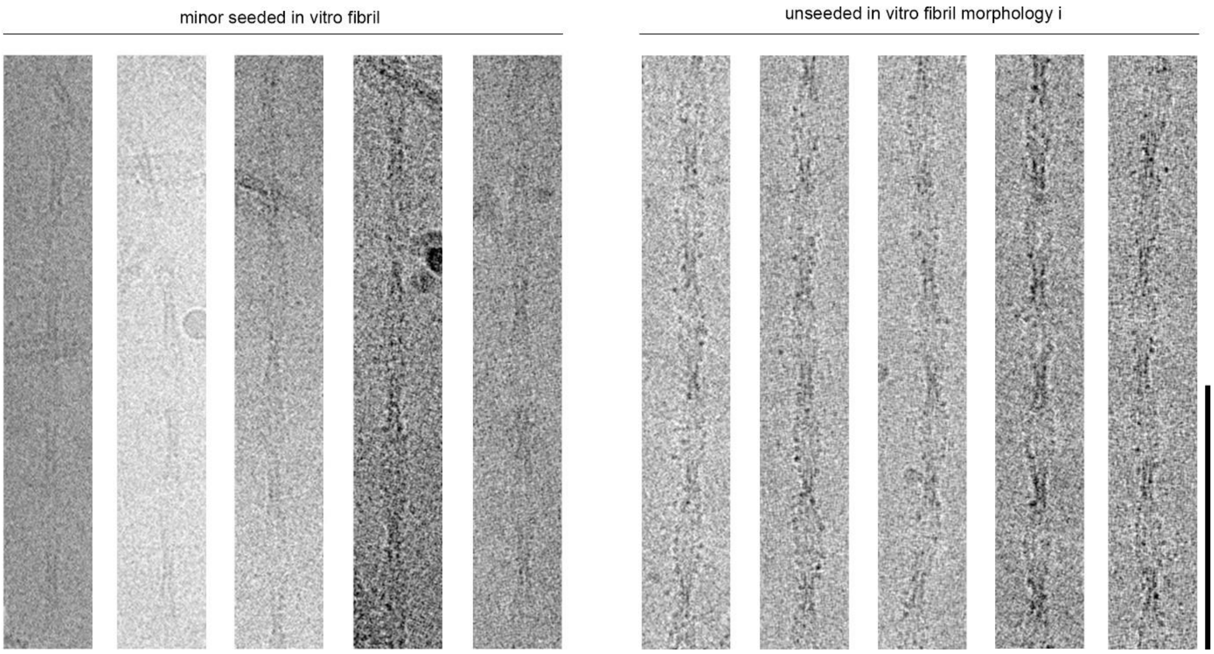

Supplementary Figure 4.

Cryo-EM images of the minor morphology of the seeded in vitro fibrils and of the unseeded in vitro fibril morphology i.

The cryo-EM images are different for the two fibrils. Scalebar: 100 nm. The micrographs are representative for 1,762 micrographs of the seeded in vitro and 3,001 of the unseeded in vitro sample.

Supplementary Figure 5

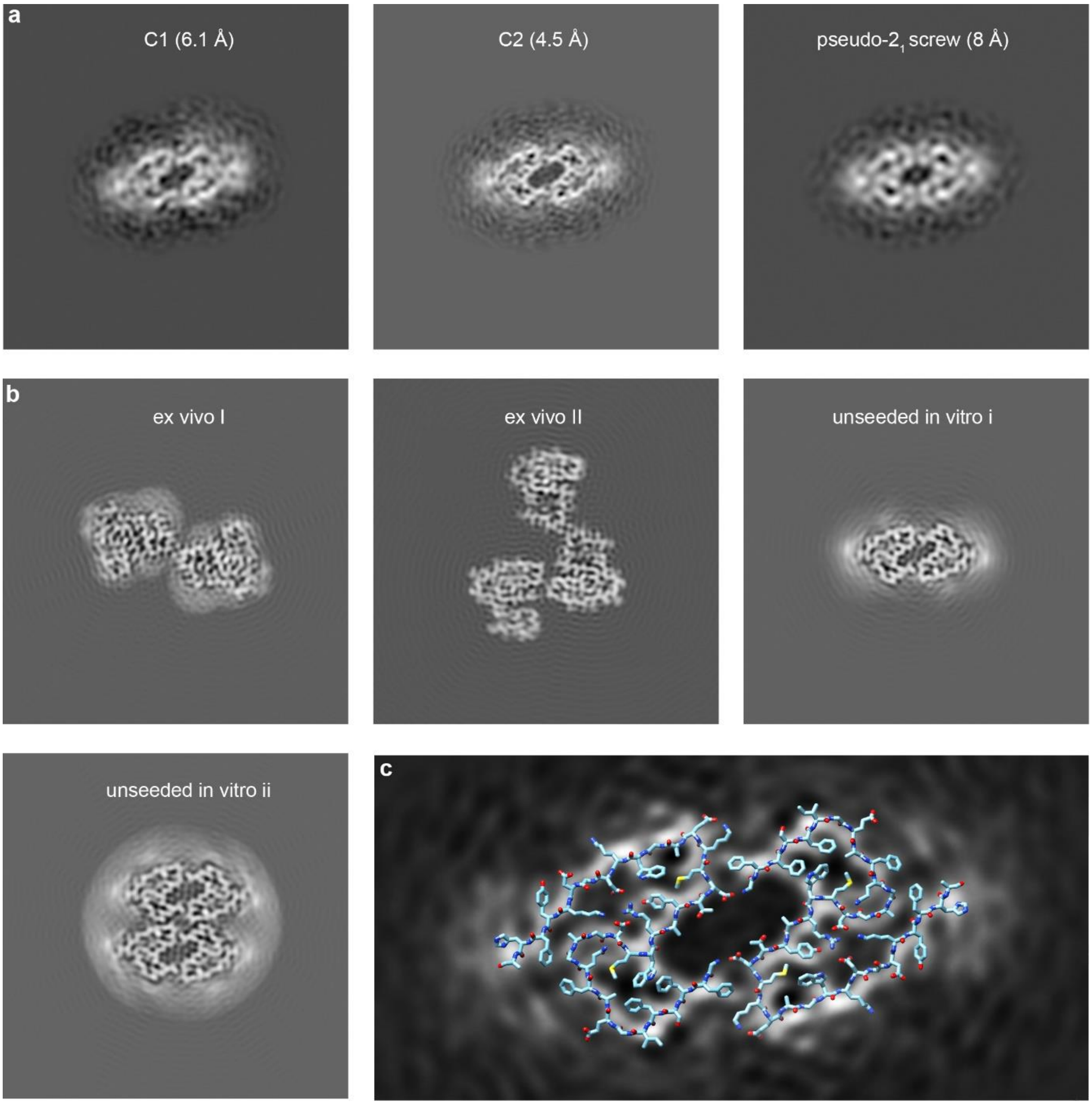

Supplementary Figure 5.

Reconstruction of the minor morphology of the seeded in vitro fibrils.

(a) 4.8 Å thick slices of 3D maps of the minor morphology that were reconstructed by imposing C1, C2 or pseudo-2_1 screw symmetry as indicated in the figure. The values in brackets provide the resolution based on the 0.143 FSC criterion. (b) 4.8 Å thick slice of 4.5 Å lowpass filtered densities of the ex vivo fibril morphologies I and II[1,2] and the unseeded in vitro fibril morphologies i and ii[2]. (c) Superimposition of the reconstructed density (C2) of the minor

morphology of the seeded in vitro fibrils with the molecular model of the unseeded in vitro fibril morphology i (PDB 6ZCF).

Supplementary Figure 6

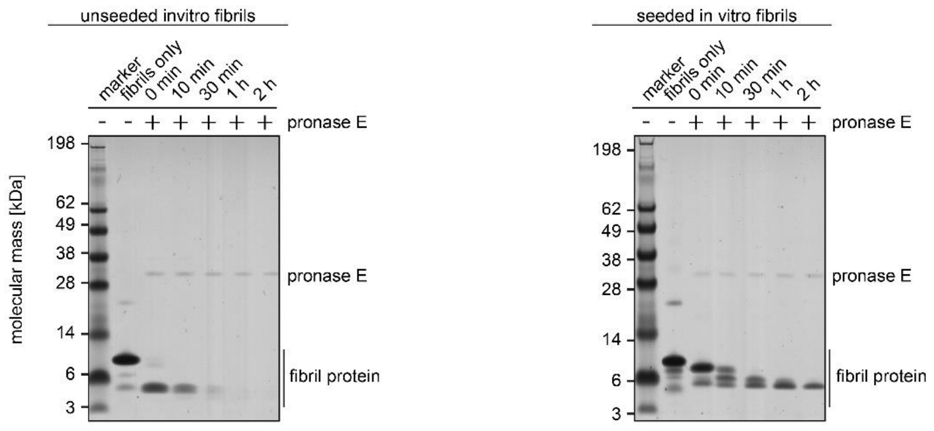

Supplementary Figure 6.

Seeded fibrils show a higher resistance to pronase E than unseeded fibrils.

Coomassie stained denaturing protein electrophoresis gels with seeded and unseeded in vitro fibrils, which were incubated with pronase E for different periods of time as indicated in the figure. The lane next to the marker shows fibrils before pronase E addition. All experiments were performed in triplicates (n=3).

Supplementary Tables

Supplementary Table 1

Microscope	Titan Krios (Thermo Fisher Scientific)
Camera	K2 Summit (Gatan)
Acceleration voltage (kV)	300
Magnification	x 130,000
Defocus range (µm)	0.2–3.5
Dose rate ($e^-/Å^2/s$)	3.35
Number of movie frames	40
Exposure time (s)	13.2
Total electron dose ($e^-/Å^2$)	40.9
Pixel size (Å)	1.04
Gatan imaging filter	20 eV
Mode	Counting mode
Box size (pixel)	300
Inter box distance (Å)	27.7
Number of extracted segments	141159
Number of segments after 2D classification	141159
Number of segments after 3D classification	107856
Resolution, 0.143 FSC criterion (Å)	2.69
Map sharpening B-Factor ($Å^2$)	-57.5
Helical rise (Å)	2.4
Helical twist (°)	179.425
Symmetry imposed	C1

Supplementary Table 1

Structural statistics of cryo-EM data collection and image processing of the major morphology of the seeded in vitro fibrils.

Supplementary Table 2

Initial model used	6DSO
Model resolution, 0.143 FSC criterion (Å)	2.6
Model composition	
Non-hydrogen atoms	6588
Protein residues	828
Ligands	0
RMSDs	
Bond length (Å)	0.017
Bond angle (°)	1.630
Validation	
Molprobity score	1.12
Clash score	1.85
Poor rotamers (%)	0
Ramachandran plot	
Favoured (%)	97.01
Allowed (%)	2.99
Disallowed (%)	0
EMRinger score	
z score	15.53
score	6.83
Map CC	
CCmask	0.79

Supplementary Table 2

Structural statistics of model building and refinement.

Supplementary References

1. Liberta, F. *et al.* Cryo-EM fibril structures from systemic AA amyloidosis reveal the species complementarity of pathological amyloids. *Nat. Commun.* **10**, 1–10 (2019).
2. Bansal, A. *et al.* AA amyloid fibrils from diseased tissue are structurally different from in vitro formed SAA fibrils. *Nat. Commun.* **12**, 1–9 (2021).
