# Supplementary material for: Cryo-EM demonstrates the in vitro proliferation of an ex vivo amyloid fibril morphology by seeding
Source: Nat Commun. 2022 Jan 10;13:85. doi: 10.1038/s41467-021-27688-5 (PMC8748726; doi:10.1038/s41467-021-27688-5)
Supplement: Supplementary file 2 — Reporting Summary [file 41467_2021_27688_MOESM2_ESM.pdf]

nature portfolio

Corresponding author(s): Marcus Fändrich

Last updated by author(s): Nov 11, 2021

Reporting Summary

Nature Portfolio wishes to improve the reproducibility of the work that we publish. This form provides structure for consistency and transparency in reporting. For further information on Nature Portfolio policies, see our Editorial Policies and the Editorial Policy Checklist.

Statistics

For all statistical analyses, confirm that the following items are present in the figure legend, table legend, main text, or Methods section.

n/a	Confirmed	
☐	☒	The exact sample size (*n*) for each experimental group/condition, given as a discrete number and unit of measurement
☐	☒	A statement on whether measurements were taken from distinct samples or whether the same sample was measured repeatedly
☒	☐	The statistical test(s) used AND whether they are one- or two-sided<br>*Only common tests should be described solely by name; describe more complex techniques in the Methods section.*
☒	☐	A description of all covariates tested
☒	☐	A description of any assumptions or corrections, such as tests of normality and adjustment for multiple comparisons
☐	☒	A full description of the statistical parameters including central tendency (e.g. means) or other basic estimates (e.g. regression coefficient) AND variation (e.g. standard deviation) or associated estimates of uncertainty (e.g. confidence intervals)
☒	☐	For null hypothesis testing, the test statistic (e.g. *F*, *t*, *r*) with confidence intervals, effect sizes, degrees of freedom and *P* value noted<br>*Give P values as exact values whenever suitable.*
☒	☐	For Bayesian analysis, information on the choice of priors and Markov chain Monte Carlo settings
☒	☐	For hierarchical and complex designs, identification of the appropriate level for tests and full reporting of outcomes
☒	☐	Estimates of effect sizes (e.g. Cohen's *d*, Pearson's *r*), indicating how they were calculated

Our web collection on statistics for biologists contains articles on many of the points above.

Software and code

Policy information about availability of computer code

Data collection	SerialEM v3.7
Data analysis	Relion v3.0.4, MotionCor v2.1, Gctf v1.06, Coot v0.8.9, PHENIX v1.16, IMOD, Fiji (ImageJ) 1.52, UCSF Chimera 1.13.1, Situs (pdbsymm) 3.1, Gromacs simulation package version 2018, VMD 1.9.3

For manuscripts utilizing custom algorithms or software that are central to the research but not yet described in published literature, software must be made available to editors and reviewers. We strongly encourage code deposition in a community repository (e.g. GitHub). See the Nature Portfolio guidelines for submitting code & software for further information.

Data

Policy information about availability of data

All manuscripts must include a data availability statement. This statement should provide the following information, where applicable:

- Accession codes, unique identifiers, or web links for publicly available datasets
- A description of any restrictions on data availability
- For clinical datasets or third party data, please ensure that the statement adheres to our policy

The reconstructed cryo-EM map was deposited in the Electron Microscopy Data Bank (EMDB) with the accession codes EMD-13089 [https://www.ebi.ac.uk/emdb/EMD-13089]. The coordinates of the fitted atomic model were deposited in the Protein Data Bank (PDB) under the accession code 7OVT [https://doi.org/10.2210/pdb7OVT/pdb]. The following previously published coordinates were used in Fig. 1: PDB 6DSO [http://dx.doi.org/10.2210/pdb6dso/pdb], 6ZCH [http://dx.doi.org/10.2210/pdb6zch/pdb], 6ZCF [http://dx.doi.org/10.2210/pdb6zcf/pdb], 6ZCG [http://dx.doi.org/10.2210/pdb6zcg/pdb]; Fig. 4: PDB 6DSO [http://dx.doi.org/10.2210/pdb6dso/pdb]; Supplementary Fig. 4: PDB 6ZCF [http://dx.doi.org/10.2210/pdb6zcf/pdb]. The source data associated with following figures has

been provided with this paper: Figs. 2, 3, 5, Supplementary Fig. 1. The data that support the findings of this study are available from the corresponding author upon reasonable request.

Field-specific reporting

Please select the one below that is the best fit for your research. If you are not sure, read the appropriate sections before making your selection.

☒ Life sciences ☐ Behavioural & social sciences ☐ Ecological, evolutionary & environmental sciences

For a reference copy of the document with all sections, see nature.com/documents/nr-reporting-summary-flat.pdf

Life sciences study design

All studies must disclose on these points even when the disclosure is negative.

Sample size	Fibrils were generated by the additon of recombinant murine SAA1.1 of ex vivo fibrils extracted from murine tissue acting as seeds. A dataset of 1,762 micrographs was recorded. These data were sufficient to lead to a decent reconstruction.
Data exclusions	Initially 141,159 segments were selected. Out of those 107,856 segments were used for the final reconstruction.
Replication	Electron microscopy data is based on a single sample. 107,856 segments were used for the reconstruction. SDS-Page data were replicated successfully in three independent experiments. Representative data is shown. ThT fibrillation kinetics were successfully repeted three times (all measurements are shown in Fig. 2a).
Randomization	The data shown represents a single case study, therefore randomization is not relevant to study.
Blinding	The data shown represents a single case study, therefore blinding is not relevant to study.

Reporting for specific materials, systems and methods

We require information from authors about some types of materials, experimental systems and methods used in many studies. Here, indicate whether each material, system or method listed is relevant to your study. If you are not sure if a list item applies to your research, read the appropriate section before selecting a response.

Materials & experimental systems

n/a	Involved in the study	
☒	☐	Antibodies
☒	☐	Eukaryotic cell lines
☒	☐	Palaeontology and archaeology
☐	☒	Animals and other organisms
☒	☐	Human research participants
☒	☐	Clinical data
☒	☐	Dual use research of concern

Methods

n/a	Involved in the study	
☒	☐	ChIP-seq
☒	☐	Flow cytometry
☒	☐	MRI-based neuroimaging

Animals and other organisms

Policy information about studies involving animals; ARRIVE guidelines recommended for reporting animal research

Laboratory animals	Femal 6- to 8-week-old NMRI mice (charles River Laboratories)
Wild animals	The study did not involve wild animals.
Field-collected samples	The study did not involve saples collected from the field
Ethics oversight	The animals were generated based on an animal experiment permission (no. 1165) from the Regirungspräsidium Tübingen.

Note that full information on the approval of the study protocol must also be provided in the manuscript.
